# Understanding in conditions of neutrality: a closer look at its objects

**DOI:** 10.1007/s44204-026-00410-2

**Published:** 2026-04-28

**Authors:** Miloud Belkoniene

**Affiliations:** 1 Philosophy Department, University of Zurich, Zurich, Switzerland; 2 African Centre for Epistemology and Philosophy of Science, University of Johannesburg, Johannesburg, South Africa

**Keywords:** Understanding, Neutrality, Inquiry, Grasping, Live options

## Abstract

In her article titled ‘A portrait of understanding as a non-factive state’, Sjölin Wirling provides an engaging ‘portrait’ of a type of understanding—termed ‘non-factive objectual understanding’—that inquirers who have yet to settle certain questions concerning a target phenomenon can achieve under particular conditions. This paper investigates whether, in the situations Sjölin Wirling discusses, the relevant epistemic improvements really are improvements in our understanding *of* phenomena.

## Introduction

In her article titled ‘A portrait of understanding as a non-factive state’, Sjölin Wirling provides an engaging ‘portrait’ of a type of understanding—termed ‘non-factive objectual understanding’—that inquirers who have yet to settle certain questions concerning a target phenomenon can achieve under particular conditions. More precisely, according to Sjölin Wirling, reasonably well-informed and competent inquirers who are not yet in a position to settle certain questions concerning a target phenomenon can gain a non-factive understanding of this phenomenon provided they have a sufficient *grasp* of a range of potential answers that they correctly recognise as *live options*.

An innovative aspect of Sjölin Wirling’s proposal is that if her portrait of understanding as a non-factive state proves to be accurate, contrary to what has been previously assumed, improvements in our understanding of phenomena do not always result from settling certain matters relating to these phenomena. However, a question that remains is whether, in the situations Sjölin Wirling discusses, the relevant epistemic improvements really are improvements in our understanding *of* phenomena.

## The portrait

When we think of understanding in relation to inquiry, it is *prima facie* plausible that an improvement in our understanding of a phenomenon depends on our being in a position to settle certain matters relating to this phenomenon. This may involve being in a position to know, or acquiring justification for believing, certain truths concerning its structure, function, or causal history. But as philosophers such as Elgin (2017) emphasise, it may also involve accepting felicitous falsehoods that put us in a position to extract relevant information about the phenomenon. Regardless, it seems that improving our understanding of a phenomenon requires *endorsing* certain representations of it—whether this amounts to accepting, partially believing, fully believing with justification, or knowing these representations—and thus acquiring new information about it.^1^ Put differently, *neutrality* regarding matters relevant to a phenomenon appears incompatible with improving our understanding of it.

According to Sjölin Wirling, however, neutrality, thus understood, is not always incompatible with an improvement in our understanding of a phenomenon. Even if an inquirer is not yet in a position to settle matters relevant to the phenomenon they are interested in and is therefore neutral regarding a range of potential accounts of this phenomenon, they can still achieve a non-factive understanding of it, where:

Non-Factive Objectual Understanding (NFOU): An epistemic subject S has non-factive objectual understanding of some target phenomenon T just in case (i) S grasps and is able to navigate a plurality of accounts of T, A_1_…A_n_; (ii) A_1_…A_n_ are live options relative to a suitably rich and accurate body of settled propositions relevant to T, and; (iii) S recognises that A_1_…A_n_ are live options with respect to T. (Sjölin Wirling, 2026, pp. 4–5)

Before turning to the three conditions on which the achievement of non-factive objectual understanding depends, consider the following example, which is supposed to exemplify the kind of situation in which such an understanding can be achieved:

Tiger bush is a form of vegetation which grows in stripes, separated by barren areas, forming a pattern reminiscent of that in the tiger’s fur. Scientists don’t know exactly what causes this self-organizing pattern formation. They construct models – Turing models, kernel models, differential flow models – that underwrite possible explanations of tiger bush, insofar as they are compatible with current scientific knowledge pertaining to the tiger bush and related phenomena. (Sjölin Wirling, 2026, p. 4)

As Sjölin Wirling outlines, multiple models of the tiger bush’s formation pattern are regarded as live options. None can be ruled out, based on the available evidence, as failing to capture the mechanisms that underwrite this formation pattern. Nevertheless, insofar as scientists have a sufficient grasp of the models they (correctly) regard as live options, they are, Sjölin Wirling argues, in a position to acquire a non-factive objectual understanding of the tiger bush’s formation pattern.

The notion of grasp used here to characterise the scientists’ epistemic standing relative to the models they regard as live options is borrowed from recent literature on the epistemology of understanding. Following philosophers such as Hills (2016), Sjölin Wirling considers that grasping an account of a phenomenon involves having cognitive control over it in the sense of being able to reason conditionally based on the information it potentially contains about the target phenomenon. For example, grasping an account of the tiger bush’s formation pattern involves, in part, being able to use this account to draw certain conclusions about possible cases where the phenomenon arises, as well as providing explanations for why the phenomenon arises in these cases.

The notion of live options, for its part, denotes a restricted type of epistemic possibility. More precisely, live options with respect to a phenomenon T are accounts of T—that is, representations of how T allegedly is—that strongly cohere with a body of epistemically privileged propositions in the sense that the body of propositions relevant to T that are regarded as *settled* supports the conclusion ‘that something along the lines of the account could be both illuminating and correct’ (Sjölin Wirling, 2026 p. 7).^2^

Condition (ii), where ‘A_1_…A_n_ are live options relative to a suitably rich and accurate body of settled propositions relevant to T’, is key, in Sjölin Wirling’s view, to seeing how a subject’s epistemic position regarding the phenomenon T can improve by way of achieving a non-factive objectual understanding of T, although this subject is not in a position to settle *new* matters relevant to T and obtain *new* information about T. For, as Sjölin Wirling suggests, the fact that the accounts of T that a subject grasps and recognises as live options are actually live options guarantees that the cognitive control the subject has over them and her ability to navigate various options with respect to T are tethered to facts. That is, it guarantees that a subject’s cognitive control over these accounts and her ability to navigate various options with respect to T are constrained by the facts relevant to T that she regards as settled.

## The objects

While it is difficult to deny that the epistemic standing of the subjects involved in the situations considered by Sjölin Wirling improves, it is unclear, given that this improvement is best captured in terms of understanding, that the understanding achieved is truly an understanding *of* the phenomenon under inquiry.

First, one may wonder if the understanding achieved by scientists who grasp the multiple models of the tiger bush’s formation pattern, yet remain neutral concerning which model actually captures the mechanisms that underwrite this pattern, does not ultimately amount to an understanding of the relevant models, rather than an understanding of the formation pattern. After all, as Sjölin Wirling acknowledges, the relevant notion of grasp plausibly denotes a form of understanding whose paradigmatic objects are accounts or theories of phenomena. That is, saying that the scientists inquiring into the tiger bush’s formation pattern have a good grasp of the models they regard as live options is to say that they have a good understanding of these models. One may therefore worry that what Sjölin Wirling characterises as a non-factive objectual understanding of the tiger bush’s formation pattern just is an understanding of the models of this phenomenon that are regarded as live options.

Of course, this worry did not escape Sjölin Wirling’s attention. While she acknowledges that grasping certain accounts of T can equally be thought of as having an understanding of these accounts, she stresses that the fact that these accounts that are also *correctly* regarded as live options makes a difference to one’s epistemic traction on T itself. For instance, the fact that the models of the tiger bush’s formation pattern that scientists understand are correctly regarded as live options makes a difference to the scientists’ epistemic traction on this formation pattern. If the models of the tiger bush’s formation pattern were merely possible accounts of this formation pattern completely unconstrained by already settled matters relevant to this phenomenon, the scientists would lack a distinctive epistemic traction on this phenomenon that is supposedly captured by the notion of non-factive objectual understanding. Of course, the question then is what is the exact nature of the relevant epistemic traction that scientists are supposed to possess?

According to Sjölin Wirling, grasping accounts of a phenomenon that one correctly regards as live options improves one’s epistemic traction on a phenomenon by *enriching* it. In particular, non-factive objectual understanding is:

At once a bird’s eye view of a map of options that one can navigate, a heightened awareness of one’s epistemic situation with respect to the phenomenon, and as a result, a more strongly felt cognitive contact with the phenomenon of interest. (Sjölin Wirling, 2026, p. 12)

Having what Sjölin Wirling characterises as ‘a bird’s eye view of a map of options that one can navigate’ plausibly amounts to having a good grasp or understanding of these options if the relevant notion of grasp is to be construed as a kind of cognitive control over its object—that is, over an account of T that one correctly regards as a live option. Yet, having a ‘heightened awareness of one’s epistemic situation with respect to the phenomenon’ cannot simply amount to having an understanding of the accounts of T that one correctly regards as live options. For, having cognitive control over these options and thus being able to navigate them is, in principle, independent from having such an appreciation of one’s epistemic situation with respect to T. One can, for example, have a good grasp of the multiple models of the tiger bush’s formation pattern in the sense of being able to use these models to make predictions and derive explanations without appreciating one’s epistemic situation with respect to this particular phenomenon.

The idea that non-factive objectual understanding involves a heightened awareness or appreciation of a subject’s epistemic situation with respect to T thus appears to be key to explaining why such an understanding does not simply amount to an understanding of the accounts of T that the subject correctly regards as live options. Yet, even if one grants that non-factive objectual understanding involves such an appreciation, it is unclear whether this can vindicate the idea that the relevant non-factive objectual understanding is an understanding of the phenomenon under inquiry. For instance, even if one grants that scientists who grasp the multiple models of the tiger bush’s formation pattern and correctly regard them as live options have a better appreciation of their epistemic situation with respect to this phenomenon, it does not follow that they have a better understanding of the tiger bush’s formation pattern itself. After all, that one better understands T does not follow from the fact that one better appreciates or understands one’s epistemic situation with respect to T. If I come to understand my current perceptual experience of the wall in front of me as being white as a veridical perceptual experience, I improve my understanding of my present epistemic situation with respect to the colour of the wall I am looking at. Yet, it does not then follow that I have improved my understanding of the colour of the wall or of the wall itself.

Thus, even if it is granted that non-factive objectual understanding involves both ‘a bird’s eye view of a map of options that one can navigate’ and ‘heightened awareness of one’s epistemic situation with respect to the phenomenon’, it appears that this epistemic standing is best conceived of as combining an understanding of one’s epistemic situation with respect to T and an understanding of the accounts of T that one correctly regards as live options. Yet, such a conception would put pressure on Sjölin Wirling’s overall project. For, given such a conception, the cases discussed by Sjölin Wirling would no longer support the conclusion that neutrality on matters relevant to T is compatible with improving one’s understanding of T. They would, at best, be evidence that one’s understanding of one’s epistemic situation with respect to T and one’s understanding of the accounts of T that one correctly regards as live options can improve without acquiring new information about T.

One way to resist this worry may be to rely on the distinction introduced by Le Bihan (2017) between understanding phenomena—the way the world appears to us—and understanding what the world is actually like. As Le Bihan notes, one way to acquire an understanding of phenomena is to acquire an understanding of how these phenomena actually arise—to acquire an understanding of some aspects of the world. Yet, she argues, acquiring modal understanding—coming to understand how a phenomenon might arise as opposed to understanding how it actually arises—can also improve our understanding of phenomena without thereby improving our understanding of the world. The reason for this is that a ‘salient feature of the phenomena is that they can arise in *many possible ways* besides the particular way in which they *actually* arise in the world’ (Le Bihan, 2017, p. 124).

Thus, it may be argued that as non-factive objectual understanding involves knowing how to navigate the possibilities related to T that one regards as live options, improving one’s non-factive objectual understanding, like improving one’s modal understanding, is a way of improving one’s understanding of *phenomena*. However, one issue with this line of response is that, as Sjölin Wirling (2026, pp. 20–21) outlines, the notion of modal understanding introduced by Le Bihan is tied to a subject’s ability to navigate the *objective* possibility space associated with a phenomenon. That is, what Le Bihan claims is that one’s understanding of phenomena can improve by settling certain matters about the objective possibility spaces associated with phenomena—that is, by acquiring information about the many possible ways they might arise besides the particular way they actually arise in the world. In contrast, the notion of non-factive objectual understanding introduced by Sjölin Wirling is supposed to denote an improvement in a subject’s epistemic standing that does not depend on one acquiring new information concerning how a phenomenon actually arises or how it might (objectively) arise. Consequently, it appears that the doubts just expressed concerning the claim that, in the situations considered by Sjölin Wirling, one’s understanding *of* a particular phenomenon improves cannot be diffused by appeal to the kind of considerations put forward by Le Bihan. What needs to be shown is that improvements in one’s understanding of T—as opposed to improvements in one’s understanding of one’s epistemic situation with respect to T or in one’s understanding of the accounts of T that one regards as live options—are compatible with remaining neutral on matters related to how T actually arises in the world *and* the many ways it might (objectively) arise.

## More objects

Even if one grants that, in the situations considered by Sjölin Wirling, the relevant improvement is best captured in terms of understanding, it is therefore unclear whether it is truly one’s understanding of the phenomenon under inquiry that improves. As I have argued, it seems that the relevant improvement can be equally thought of as an improvement in one’s understanding of one’s epistemic situation with respect to the target phenomenon and in one’s understanding of the accounts that one regards as live options. However, another case offered by Sjölin Wirling may be worth considering at this stage. Discussing the current philosophical debate concerning the metaphysics of properties and the various accounts offered in its context, Sjölin Wirling submits that:

Philosophers are not in a position to justifiably say which of these (if any) is the correct account, but several of them are regarded as live options in the sense that they draw on and are constrained by what we take ourselves to know about the phenomena in question: from science, from linguistics, from ordinary experience, and so on. Yet, I suggest that an epistemic subject who grasps a range of these different accounts and recognise that they are all live options has a certain understanding *of this cluster of phenomena*, and this is epistemically valuable independently of whether or not we can expect such a grasp to be conducive to ever settling the issue of whether there are any properties and if so, what their nature is. (Sjölin Wirling, 2026, pp. 3–4)

This case is particularly interesting when it comes to Sjölin Wirling’s main point—that improving one’s understanding of a phenomenon or a cluster of phenomena is compatible with neutrality—as it relates to broader metaphilosophical concerns. Observing that philosophical inquiry is characterised by widespread and seemingly unresolvable disagreements among philosophers concerning central philosophical questions, philosophers such as Van Inwagen (2004), Dietrich (2010), and Chalmers (2015) have indeed expressed doubt concerning the existence of genuine philosophical progress—at least compared to other fields of inquiry. For if philosophy, like other fields of inquiry, primarily seeks to secure knowledge or collective knowledge of the true answers to central philosophical questions, then the pervasiveness of disagreement over such questions may be taken to indicate that, so far, little progress, if any, has been made towards that goal and that no such progress should be expected in the future.

However, if Sjölin Wirling is correct about the understanding that philosophers have of the metaphysics of properties as a result of grasping the competing accounts of this cluster of phenomena and correctly regarding them as live options, it seems that this pessimism about philosophical progress based on the pervasiveness of disagreement over central philosophical questions is misplaced. After all, non-factive objectual understanding is supposed to have final epistemic value, and if philosophers can achieve such a finally valuable standing without settling new matters relevant to the metaphysics of properties, then there can be genuine progress in philosophy even if disagreement over central philosophical questions persists. The relevant notion of progress simply needs to be reconceived in relation to other finally valuable epistemic standings, such as non-factive objectual understanding.

Pessimists such as Chalmers appear to have considered dialectical moves in the vicinity of that just envisioned:

It is sometimes said that an obsession with truth reflects an overly scientistic conception of philosophy. We should not think of philosophy as a quest for the answers. Instead it is a quest for something else: understanding, clarity, enlightenment. I agree that these are goals worth pursuing, and that philosophy can help us pursue them. And I can see why, in the absence of answers to philosophical puzzles, it might seem especially appealing to focus on these goals instead. Still, I think we should acknowledge that this reaction involves something of a lowering of our sights for philosophy. (Chalmers, 2015, p. 14)

Now, one reason to think that such a reaction involves ‘a lowering of our sights for philosophy’ is that one may insist on distinguishing improvements in our understanding of central philosophical questions from improvements in our understanding of the phenomena, or cluster of phenomena, these questions are about. While being able to navigate—thanks to one’s grasp—the various competing accounts of the metaphysics of properties and having a heightened awareness of one’s epistemic situation with respect to this cluster of phenomena can plausibly be construed as an improvement in one’s understanding of the *questions* and *problems* these accounts are designed to address, it is unclear that it constitutes an improvement in one’s understanding *of* the metaphysics of properties itself. For that one better understands the metaphysics of properties itself does not follow from the fact that one better understands central philosophical questions and problems concerning the metaphysics of properties. Better understanding the latter, one may insist, typically involves answering the questions and problems that one understands as a result of grasping the available accounts of the metaphysics of properties and correctly regarding them as live options.

## Conclusion

While, in my view, the portrait of non-factive objectual understanding provided by Sjölin Wirling falls short of showing that improvements in our understanding of certain phenomena are compatible with remaining neutral about matters relevant to these phenomena, it raises interesting and important questions concerning the object of the understanding that appears to be gained in the situations discussed by Sjölin Wirling. I have outlined that the understanding achieved in these situations is equally well—and more plausibly—construed as an understanding of the accounts that are correctly regarded as live options, as an understanding of one’s epistemic situation with respect to the phenomena these accounts represent, or as an understanding of the questions and problems these accounts are designed to address. However, the precise relations that exist between these different understandings—theoretical understanding, understanding of one’s own epistemic situation, and understanding of certain questions and problems—remain unclear, and I believe that one significant merit of the portrait provided by Sjölin Wirling is that it goes some way towards shedding light on these relations.

## Data Availability

Not applicable.

## Footnotes

1 On the claim that understanding does not necessarily involve belief, see Elgin (2004, 2017), Dellsén (2017, 2018, 2021), and Wilkenfeld (2017). According to these philosophers, acceptance or partial belief is sufficient. See Belkoniene (2022) and Kelp and Belkoniene (2026) for a defence of the claim that understanding requires full belief with justification. See also Sliwa (2015), Kelp (2015, 2017), and Khalifa (2017) for versions of the claim that understanding requires knowledge.

2 Note that Sjölin Wirling often suggests that the relevant body of epistemically privileged propositions is not determined by what one regards as settled about T, in the sense of merely believing it to be true. In fact, the notion of propositions that are known to be true of T is often used to characterise this body of propositions.
